# The Dynamics, Causes and Possible Prevention of Hepatitis *E* Outbreaks

**DOI:** 10.1371/journal.pone.0041135

**Published:** 2012-07-24

**Authors:** Betty Nannyonga, David J. T. Sumpter, Joseph Y. T. Mugisha, Livingstone S. Luboobi

**Affiliations:** 1 Department of Mathematics, Makerere University, Kampala, Uganda; 2 Mathematics Department, Uppsala University, Uppsala, Sweden; Centers for Disease Control and Prevention, United States of America

## Abstract

Rapidly spreading infectious diseases are a serious risk to public health. The dynamics and the factors causing outbreaks of these diseases can be better understood using mathematical models, which are fit to data. Here we investigate the dynamics of a Hepatitis *E* outbreak in the Kitgum region of northern Uganda during 2007 to 2009. First, we use the data to determine that 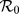 is approximately 2.25 for the outbreak. Secondly, we use a model to estimate that the critical level of latrine and bore hole coverages needed to eradicate the epidemic is at least 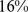 and 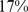 respectively. Lastly, we further investigate the relationship between the co-infection factor for malaria and Hepatitis *E* on the value of 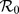 for Hepatitis *E*. Taken together, these results provide us with a better understanding of the dynamics and possible causes of Hepatitis *E* outbreaks.

## Introduction

Outbreaks of diseases such as avian influenza, SARS and West Nile Virus have alerted us to the potentially grave public health threat from emerging and re-emerging pathogens [1]–[3]. Many important infectious diseases persist on a knife-edge: rapid rates of transmission coupled with brief infectious periods. Such violent epidemic behavior has been observed in plague [4], cholera [5], pertussis [6] and more recently Hepatitis *E*. The recent outbreak of Hepatitis *E* in northern Uganda, has left many dead and a number of infectives that continue to spread the infection [7]. Hepatitis *E* is caused by infection with the Hepatitis *E* virus (HEV) which has a fecal-oral transmission route. It is a self-limiting disease but occasionally develops into an acute severe liver disease. As emerging and re-emerging infectious diseases increase in outbreak frequency, there is a compelling interest in understanding their dynamics [8]–[10].

The Kitgum outbreak, which we study here, has been linked to contaminated water or food supplies [11]. An assessment conducted by the Uganda Red Cross and district representatives in Agoro revealed that for a population of about 28,045 with 6,039 households mainly living in camps for internally displaced people in Potika as well as Agoro and Oboko satellite camps, the latrine coverage was as low as 3.7%. This means that there is one latrine for every 27 people. Further, only 23 boreholes were functional implying that the bore hole coverage is 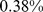, or one bore hole per 263 households.

Another possible factor that could be implicated in the outbreak of Hepatitis *E* is its possible relationship with malaria. Malaria has been shown to disarm the immune system and increase susceptibility to viral infections such as HIV [11]. Recently, in a 3-month follow-up study the pattern of co-infection of *Plasmodium falciparum* malaria and acute Hepatitis *A (HAV)*, in 222 Kenyan children under the age of 5 years was observed [12]. The incidence of HAV infections during *P. falciparum* malaria was found to be 6.3 times higher than the cumulative incidence of *HAV*, suggesting that co-infection of the two pathogens may result from changes in host susceptibility. There is also evidence both for [13], [14] and against [15], [16] an association between Hepatitis B viruses and malaria. HEV transmission route is similar to the Hepatitis A virus and thus for HEV it is important to consider possible links to co-infection with malaria. This can be done using mathematical models of multiple pathogens [17]–[22].

In this paper, mathematical models are used to study the effects of both environmental conditions and malaria on Hepatitis *E* infections. The models designed are fit to data from the Kitgum outbreak, to estimate the basic reproduction number and to relate them to the level of contamination of the environment. We assume that the small number of latrines [23], leads to contamination of environment. This in turn leads to contaminated water. Owing to the few number of bore holes in the region, lack of access to clean water gives rise to the viral infection of Hepatitis *E*.

## Results

We formulate two mathematical models: one for Hepatitis *E*-only and another for the co-infection with malaria, based on prior work as in [4], [24], [25]. In their framework, an individual is categorized according to their infection status and passes sequentially through the series of non infectious, infectious and recovered classes. A system of ordinary differential equations are then designed, analyzed and later fit to data from the Hepatitis *E* outbreak in Kitgum district to estimate desired parameters.

### Hepatitis *E* Model

First we model the epidemiology of Hepatitis *E*, an environmentally transmitted viral infection. The dynamics of the disease are an *SEIR* framework, i.e. Susceptible, Exposed, Infectious and Recovered. Hepatitis *E* virus is mainly spread by the fecal-oral route. This results either from directly touching the contaminated environment and eat without washing hands, or drinking contaminated water. In Kitgum district Uganda, most people live in internally displaced camps. The number of latrines in the area are not enough for the entire population, [11], and people use the local environment for this purpose. When rain falls, it washes the faeces into water bodies. In the Kitgum region, few people have access to clean bore hole water [11], and therefore collect water from the contaminated water sources. To model this phenomenon we use *l*, to denote the proportion of households in Kitgum with access to latrines. Therefore, the rate of change of contamination *c* of the environment is given by
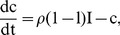
(1)where 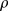 is the transmission rate of *HEV* from the infected human, *I*, to the environment.

In the human population, susceptibles, *S*, are recruited at a rate 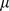 that equals to the per capita natural mortality rate for each group. This assumption is made to keep the population constant, while keeping a turnover of individuals in the population. We assume that a fraction *b* of the population has access to clean bore hole water and cannot become infected. Susceptible individuals without bore hole access become infected with the Hepatitis *E* virus at a rate 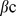, where 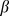 is the transmission rate of *HEV* from the contaminated environment *c*, to the human. This gives
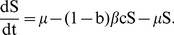
(2)


After successful infection, the individual is now exposed to *HEV* and moves to the exposed class *E*. The incubation period takes a mean period of 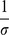 days. The equation for this group is given by
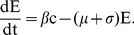
(3)


At the end of the incubation period 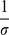, the individual becomes infectious and moves to group *I*. At this point, they display signs and symptoms that include fever, fatigue, loss of appetite, nausea, vomiting, abdominal pain, jaundice, dark urine, clay-colored stool and joint pain [26]. The infected individual may recover at a rate 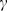. These dynamics are given by
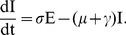
(4)


Of the total infected individuals, a fraction 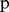 of them die due to the infection, and 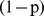 recover to join the immune group 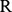. This implies that
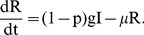
(5)


Equations (1) to (5) provide a system of equations defining the transmission of *HEV* between a contaminated environment and humans. Assuming that the dynamics of the environment are fast. This means that the environment reaches a steady-state before the humans. The quasi-stationary-state *(QSS)* for *c* can be obtained from equation (1) to give
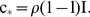
(6)


This steady state is then substituted in the human equations to give a reduced system of equations as follows:
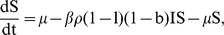


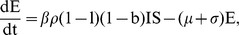


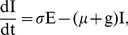
(7)

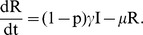
As in [27], [28], in this system, 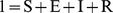. Thus, the last equation in (7) is redundant. This system of equations will be analyzed and fit to data.

The endemic stationary state is given by
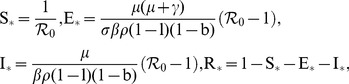
(8)where
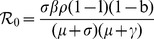
(9)is the basic reproduction number for HEV. The term 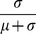 is the proportion of the exposed humans that survive the incubation period. The other fraction, 
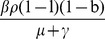
 is transmission rate of HEV during the infectious period of the human.

The disease-free equilibrium point is stable if 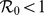 (see Supporting Information S1) When 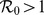 the endemic equilibrium point in equation (8) exists and is stable. This equilibrium is attained via oscillatory dynamics, with period 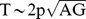, where 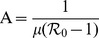 is the mean age at infection, and 
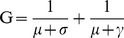
 is the ecological generation length of the infection.

**Figure 1:**
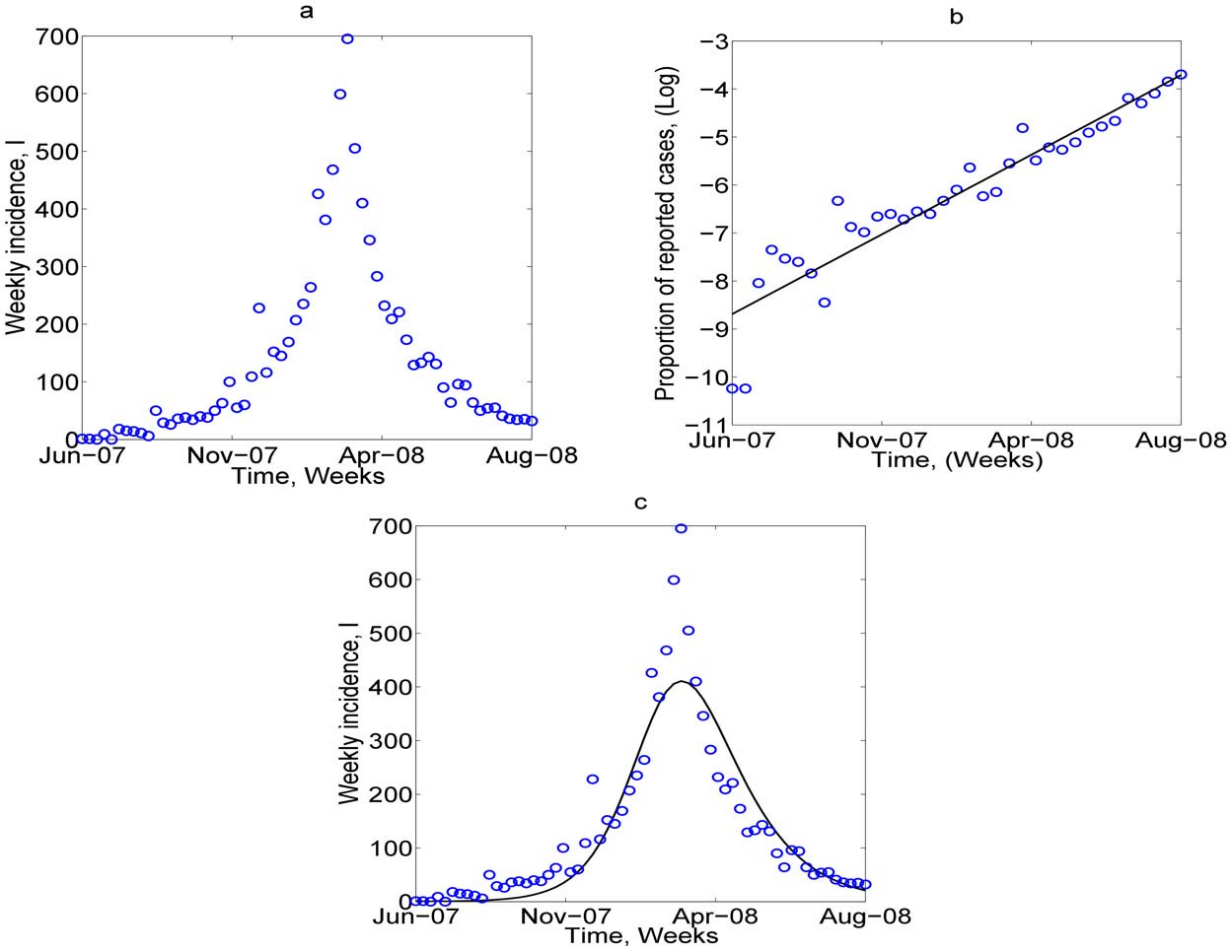
In this figure, we plot the data from the Kitgum outbreak and show how it was fit to the model. In (a), we plot the data, which is then fit using (log-)linear regression shown in (b), and then, using parameters from the PottersWheel fitting tool, we run the model again and this is shown in (c) The parameters used are 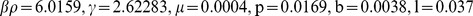.

Figure 1a is a plot of the data for the outbreak from 2007 through 2009. To estimate model parameters and determine the critical level of control needed to eradicate the epidemic, the model described by the equations in (7) is fit to the data collected during the Kitgum outbreak (Figure 1) During the invasion phase of *HEV,* the prevalence is approximately
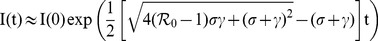
(10)


Taking the log of both sides of equation (10) and performing linear regression (details in Supporting Information S1) on this equation, (Figure 1b), gives 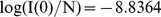. This implies that the initial number of infectives, 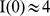. In addition, 


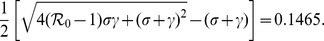
 Substituting in known parameter values for the Kitgum region 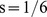, 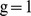 gives an estimated value 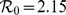.

To determine 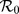 when natural mortality is not equal to zero, (i.e. 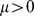), we use a non-linear differential equation fitting tool, called the PottersWheel Toolbox [29]. In this fitting technique, the 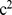 value of the sum of the squares of the differences between the observed and fitted values is minimized by searching through different parameter values. We set 
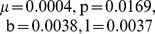
, and fit the free parameters, 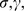 and the force of transmission 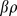. We repeat this process 50 times to produce a range of best fits. The basic reproduction number for each run is then calculated using the expression in equation (9) The fitted parameters estimate the basic reproduction number 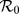 between 2.08- 2.39 with average 2.25. This value is similar to that found from the linear regression fitting. Figure 1c is a plot of the model outcome using the parameters generated from the fitting to the Kitgum outbreak.

### The Co-infection Model

In addition to Hepatitis *E*, individuals in the Kitgum region were at a risk of acquiring malaria which is endemic to Uganda. To model possible co-infection we adopt the model to include a susceptible group which comprises both those with and without malaria. That is, the total susceptible population *S′ = S+M* where *M* is the proportion of individuals infected with malaria. The malaria dynamics will not be modelled in detail here but an assumption is made that malaria continuously invades the population, and individuals move back and forth between infection and recovery from the disease. This implies that
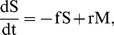


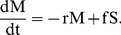
(11)The equilibrium state for this model is given by
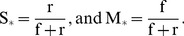
(12)Clearly, this assumption provides a much simplified model when compared to a full model of vector-borne malaria [3], [12], [27]. Our concern here, however, is how background levels of malaria effect transmission dynamics of HEV. In Kitgum, at its lowest point during March 2009, 2,316 cases of malaria were reported out of a total population of 28,045 [7]. Thus 8.3% of the population are infected with malaria at any time, 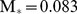. Recovery rate for malaria is 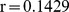 per week, and thus we set 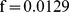.

**Figure 2:**
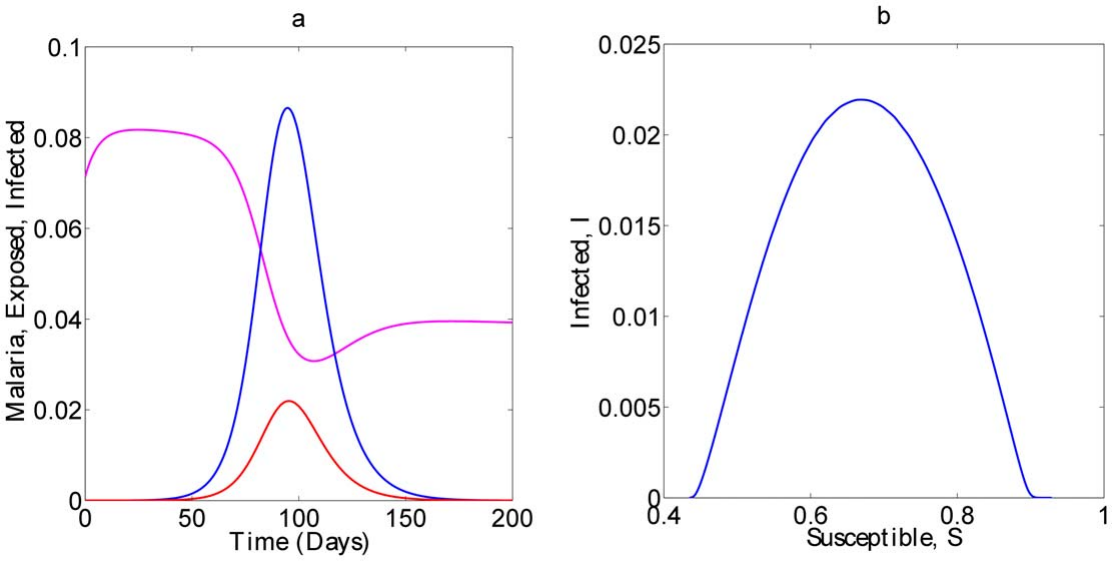
Evolutin of infection with time: Malaria infected, *M*, are represented by the magenta line, the Exposed, *E*, by blue, and the Infected, *I* by the red line. Figure (b) shows the phase space portrait in the *S-I* plane. 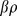 is 2, 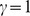, and 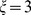. Other parameter values are given in Table 2.

Under the above assumption, equations (7) are rewritten to incorporate the malaria dynamics in equation (11) as follows:
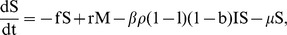


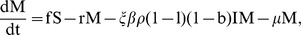


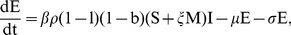
(13)

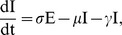


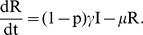
where 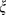 is a parameter that models change the increase (or decrease) in susceptibility to Hepatitis *E* of malaria infected individuals [12]. The other parameters are as defined in equations (7) and remain as defined there. We assume here that after exposure to *HEV*, both the susceptible and malaria infected groups join the exposed, *E* and subsequently the *I* group. In other words, individuals that harbor both infections are assumed to develop *HEV* symptoms at the same speed as those with only *HEV*. The dynamics of this model for standard parameter values are shown in Figure 2.

Using the next generation method as in van den Driessche and Watmough (2002), [30], the basic reproduction number for Hepatitis *E* in presence of malaria is given by
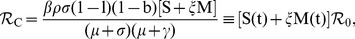
(14)where 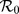 is as defined in equation (9) When 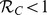 infected individuals will have more chances of recovery than of transmitting the disease further hence the epidemic will die out. When 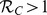, there exists an endemic equilibrium point as shown in Supporting Information S2 given by



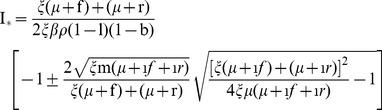
(15)If 
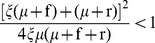
, then the roots of the quadratic equation in 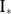 are complex conjugates and of the form *a+bi*, where 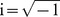. This implies that the endemic stationary point is attained via damped oscillations. The stability of this point would depend on the sign of the real part, *a*. If *a*>0, then the steady state is an unstable spiral, otherwise, it is a stable spiral. If 
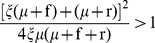
, then we have real roots, and stability of this equilibrium state will depend on the signs of these roots. If both are positive, the steady state is an unstable node; if both are negative, it is a stable node. If one of them is positive and the other negative, the steady state is a saddle point. Figure 2a shows the evolution of the malaria infected, *M*, the exposed, *E*, and the infected, *I* with time, while Figure 2b is a phase space portrait in the *SI* plane.

**Figure 3:**
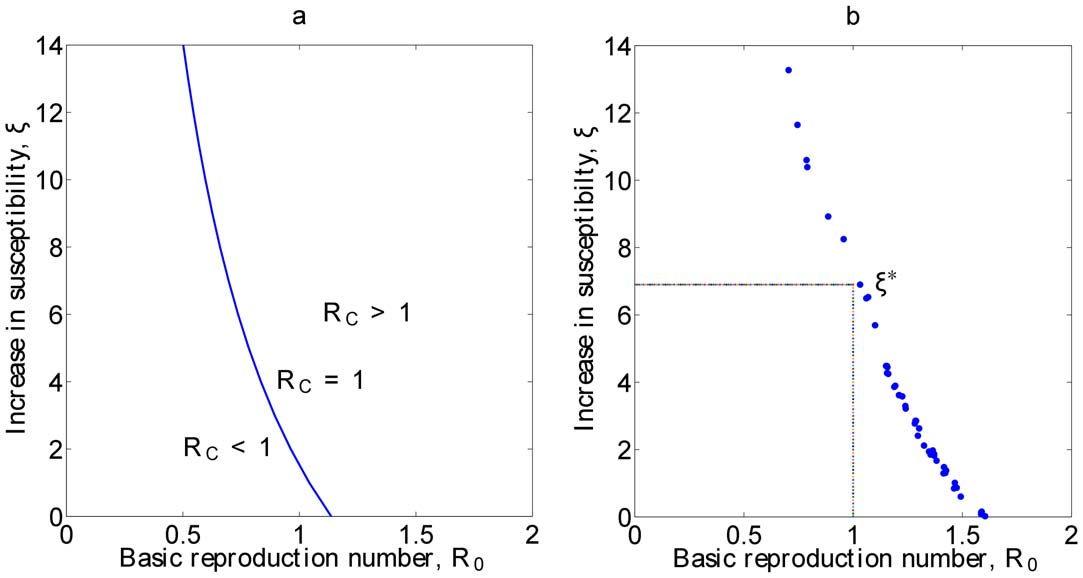
In this figure, we show the analytical calculation of 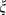 against 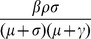 in (a), with assumption that 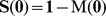, 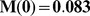, 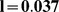 and 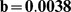. The plot of the transmission rate, 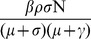 against increase in susceptibility to Hepatitis *E* of malaria infected individuals is shown in (b), 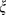, using parameters estimated from fitting tool.

From equation (14) it can be seen that the value for 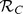 is determined by the proportions of susceptibles and malaria infectives in the population. Rearranging and assuming that 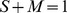 this equation gives a criteria for an epidemic of
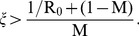
(16)This criteria is plotted in Figure 3a. As expected, if 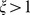 then presence of malaria increases the probability of an outbreak of Hepatitis *E*, while if 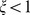 the presence of malaria inhibits Hepatitis *E*.

Assuming that malaria is at equilibrium in the population. (For example week of March 2009, with 2,316 malaria cases), gives 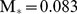, then equation (16) gives a direct relation between 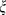 and 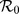. In fitting the model we note that the transmission rates 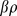 and 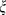 are not independent. Indeed, using PottersWheel to fit the co-infection model shows that the range of values for 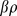 is between 1.28–4.69, 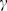 between 1.01–1.75 and 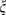 values are between 0.02–13.27 (Figure 3b) All of these values fall on line corresponding to 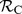 between 2.19–2.48. This relationship follows the same curve as the analytical results in Figure 3a.

To test potential interaction between Hepatitis *E* and malaria empirically, we now assume that in the absence of malaria, Hepatitis *E* has 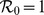 and does not spread. Thus malaria is required for the spread of Hepatitis and 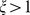. Since 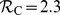 from the data and 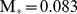, then substituting these values in to equation 14 gives 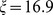. This implies that, under the assumption of co-infection as the factor which promotes Hepatitis *E*, malaria infected individuals were infected with Hepatitis *E* up to 16.9 times more than those not infected with malaria. As we gain more information about the role of co-infection, this relationship can be used to improve estimation of 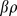.

As in [25], [27], the criterion under which Hepatitis *E* will invade the population when malaria is endemic is derived in the Supporting Information S3. Thus, Hepatitis *E* virus invades if
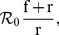
(17)and the co-infection persists if



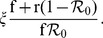
(18)As in the Hepatitis *E*-only model, PottersWheel Toolbox is used to investigate the basic reproduction number 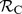, when natural mortality is not equal to zero, (i.e. 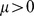) A sequence of parameter estimates are generated, this time setting the fits in sequence to 20. The process is repeated until a set of 50 readings is obtained. The parameters are chosen in such a way that 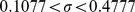 (i.e. 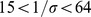 days, the Hepatitis *E* incubation period, [26], [31], [32]), and 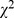 value is less than 65. The basic reproduction number for each run is calculated using the expression in equation (14).

### Cost Effective and Decision Support Analysis

The Global Burden of Disease *(GBD)* concept, first published in 1996, constituted the most comprehensive and consistent set of estimates of mortality and morbidity yet produced [33]. A *GBD* study aims to quantify the burden of premature mortality and disability for major diseases or disease groups, and uses a summary measure of population health, the *DALY* (Disability-Adjusted Life Years), to combine estimates of the years of life lost and years lived with disabilities. A *DALY* is defined as an indicator to quantify the burden of the disease and the functional limitation and premature mortality [34]. It can be used across cultures to measure health gaps as opposed to health expectancies, and the difference between a current and an ideal situation where everyone lives up to the age of the standard life expectancy, and in perfect health. In developing the *DALY* indicator, Murray and Lopez (1996), [33] identified two key value choices: (1) How long should people in good health expect to live? (2) How should we compare years of life lost through death, with years lived with poor health or disability of various levels of severity?

### Calculating the *DALYs* for Kitgum Outbreak

Since the*DALY* combines in one measure the time lived with disability, *YLD*, and the time lost due to premature mortality, *YLL*, then
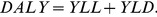
(19)


The *YLL* metric essentially corresponds to the number of deaths, 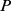, multiplied by the standard life expectancy, *L*, at the age at which death occurs. Therefore,
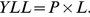
(20)To estimate *YLD* on a population basis, the number of disability cases is multiplied by the average duration of the disease and a weight factor that reflects the severity of the disease on a scale from 0 (perfect health) to 1 (dead) The basic formula (without applying social preferences) for one disabling event is given by
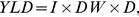
(21)where I is the number of incidence cases, 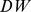 is the disability weight, and 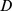 is the average duration of disability.

Since the reported cases are not specified according to age, the estimate will be done on a population basis. Typical symptoms of Hepatitis *E* include jaundice (yellow discoloration of the skin and sclera of the eyes, dark urine and pale stools), anorexia (loss of appetite), an enlarged, tender liver (hepatomegaly), abdominal pain and tenderness, nausea and vomiting, and fever and the disease may range in severity from sub-clinical to fulminant [35]. To calculate the *YLD*, we will set the disability weight to that for a diarrhea disease episode, (equal to 0.11, [33]), in untreated or treated form.

In developing the *DALY* indicator, additional social choices are taken into account. For example, is a year of healthy life gained now worth more to society than a year of healthy life gained sometime in the future? The *DALY* is an incidence-based measure, rather than a prevalence-based measure. Therefore, to estimate the net present value of years of life lost, a time discount rate to years of life lost in the future is applied, to adjust both costs and health outcomes [36]. Discounting health with time reflects the social preference of a healthy year now, rather than in the future. To do this, the value of a year of life is generally decreased annually by a fixed percentage, *d*. Therefore, equations (20) and (21) are respectively transformed to
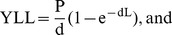
(22)

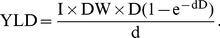
(23)According the WHO [35], the life expectancy for a Ugandan male is 51 and 48 for a female. An average of 50 years will be used. The highest number of reported cases were in the age ranges of 0–44 [23], with average of 22. Further, the total number of deaths due to Hepatitis *E* in Kitgum as of June 28 2009 were 160. We now calculate the *DALY* for the Hepatitis *E* outbreak in Kitgum district between 2007 and 2009.

From Table 1, for each 1000 individuals in Kitgum, the *YLL* were 108 and the *YLD* equaled to 144. This gave the *DALYs* of 252 per 1000.

**Table 1:** Calculating the *DALY* using the *YLL* and the *YLD*.

*YLL*	–	–	–	–	–	–
Population	*P*	*P*/1,000	Averageage atdeath	*L*	*YLL*	*YLL*/1,000
28,045	160	5.71	22	28	3,030.88	108.07
***YLD***	**–**	**–**	**–**	**–**	**–**	**–**
**Population**	***I***	***I*** **/1000**	***D*** ** (years)**	***DW***	***YLD***	***YLD*** **/1000**
28,045	9,449	336.92	2	0.11	4,035.29	143.89
*DALY* = *YLL*+*YLD*	–	–	–	–	*DALY*	*DALY*/1000

The calculation of the *DALY* using parameters from Kitgum. Parameters are as defined in text.

### Latrines, Boreholes and Education

The number of latrines and boreholes that would have prevented the Hepatitis *E* outbreak in Kitgum are calculated using our results in preceding sections. First, it is assumed that if the people had the necessary and sufficient number of latrines in addition to safe drinking, then the outbreak would not have occurred. Then, the costs of constructing the required latrines and boreholes are computed. From the results, the cost of saving one life from Hepatitis *E*, for one year is determined.

The current number of latrines in Kitgum is 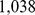 [11]. This implies one latrine per 27 people. According to the rules and regulations of Kampala City Council Authority, Building Inspection department, 1 latrine should be shared by a maximum of 5 people. We can use our estimated values of the basic reproduction number 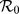 to determine the level of *l* and *b* that make 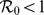. First, we use equation (9), the parameters in Table 2, and the value of 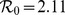, (linear regression) Assume that contamination is due to insufficient latrines. Then, for 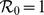, this method estimates that the latrine coverage should be increased to at least 17.1%. This translates into increasing the number of latrines from 1,038 to 4,796 (i.e. 3,756 extra latrines): 1 latrine per 7 people. Similarly, the boreholes should be increased from 23 to 230, that is, 17.7%, or 1 bore hole per 26 households. Similar results are obtained using 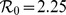 found from the non-linear fitting tool. In this case of Hepatitis *E*-only, the latrines should be increased to 16.1% and boreholes must cover 16.6% of the population. From the co-infection model, latrines should be increased to 17.5%: 4,908, (3,870 extra), 1 for 6 people. Boreholes should be increased to 18.1%, a total of 234, or 1 bore hole per 26 households.

**Table 2:** Realistic parameters for Kitgum district.

Parameter	Description	Value	Reference
*μ*	Per capita death rate of humans	0.0004/person/day	[35]
*γ*	Per capita rate of recovery from *HEV*	0.0238–0.1429/day	[11]
*p*	The proportion that died during the outbreak	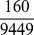	[23]
*β*	Transmission rate: human to environment	–	Estimated
*ρ*	Transmission rate: environment to human	–	Estimated
*ξ*	Moderation parameter for susceptibility to *HEV*	–	–
–	If malaria promotes *HEV*	1>	Estimated
–	If malaria inhibits *HEV*	<1	Estimated
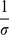	The latent period of *HEV*	15 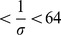 /day	[26], [32]
*l*	The proportion of humans with latrines	3.7%	[11]
*b*	The proportion of humans with bore hole water	0.38%	[11]
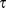	Modification parameter for the recovery period	–	–
–	of dually infected	0<τ<1	Estimated
*ε*	Modification parameter for the incubation period	–	–
–	of dually infected	0<*ε*<1	Estimated
*r*	Recovery rate from malaria by humans	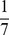 /day	[27]
*f*	Infection rate of malaria in humans	0.0129	Estimated
*d*	Real discount rate to adjust both costs	–	–
	and health outcomes	3%	[36]

Table showing parameters of the model with their description, values and reference.

**Table 3:** Minimum cost for educating the IDC dwellers by twenty (20) Counsellors.

Item	Cost per Item (USD)	No. of Days/Nights	Total cost (USD)
Hotel	50	30 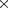 20	30,000
Meals	50	30 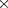 2 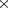 20	60,000
Transport to, within and from the Camp	20	30 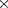 20	12,000
Transport to and from Kitgum	100	20	2,000
Total Costs	–	–	104,000

Table for estimates of educational costs over a month’s period.

Our model suggests that to eradicate the epidemic, the minimum number of additional latrines required is 3,47. The average cost of digging and constructing a basic pit latrine is approximately USD 250.00 (quotation from city council official) Therefore, 3,477 would cost a total of USD 869,250.00. Thus, the cost per disability adjusted life year averted in Kitgum, in the case of Hepatitis *E* is 869,250/7,066 = USD 123.00.

In addition to improving hygiene we should consider education. Let us now consider the case of education to the camp dwellers. Taking the simplest and cheapest scenario of hiring twenty (20) guidance and counseling officials to educate the dwellers about hepatitis *E* for about a month (that is 30) days, moving around the camp. Let us assign each counselor, 10 households per day. We then calculate the total amount in USD that would facilitate such an exercise as shown in Table 3. From the calculations, it is seen that 104,000 USD would be required. Assuming the success of such an operation this translates into 104,000/7,066 = USD 14.71 cost per disability adjusted life year.

## Discussion

The epidemic of HEV in Kitgum lasted a period of over two years [7]. Within this period, 160 individuals have lost their lives. As a result, the disease burden, the functional limitation and premature mortality have equaled a disability adjusted life years equal to 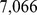, even allowing for the relatively low life expectancy in this part of Uganda. This paper provides a case study of how a simple epidemic model can be fit to such an outbreak disease. Two fitting methods have been used; the first, an analytical method and the other based on a freely available fitting tool. Using these methods, a reliable estimate of 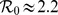 has been provided.

We then use the model to find the measures to keep 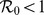. The necessary levels of latrine and bore hole coverages needed to eradicate the epidemic are both around 16 to 18%. Although the cost of construction of the required number of latrines is a one off cost, the benefits are large. Here we show what the benefits would have been in terms of protection against Hepatitis *E*. However, other diseases due to poor sanitation that have been reported in Uganda, such as cholera and dysentery, could be prevented in the same way [7].

We have also considered co-infection with malaria. If we assume that presence of malaria during a Hepatitis *E* outbreak increases persistence infection, then we estimate that a malaria infective can be infected with Hepatitis *E* up to 16 times more than one without malaria. The critical value of 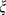 determined in this study agrees with prior studies showing increased susceptibility to other infections for malaria infected individuals [37]. However, this last result is speculative and the more important point is the relationship given in Figure 3 between co-infection and other model parameters.

## Supporting Information

Supporting Information S1 **Linear regression on system 7.**
(TEX)

Supporting Information S2 **The endemic stationary points for the co-infection model.**
(TEX)

Supporting Information S3 **The invasability criterion for the co-infection model.**
(TEX)
